# Editorial: The arts combined: cognitive, neural, and evolutionary connections among the arts

**DOI:** 10.3389/fpsyg.2026.1952382

**Published:** 2026-09-10

**Authors:** Anja-Xiaoxing Cui, Steven Brown, Gunter Kreutz

**Affiliations:** 1 Department of Musicology, University of Vienna, Vienna, Austria; 2 Department of Psychology, Neuroscience and Behaviour, McMaster University, Hamilton, ON, Canada; 3 Institute for Music, University of Oldenburg, Oldenburg, Germany

**Keywords:** arts psychology, audiovisual integration, cross-arts research, dance research, music language interaction, performing arts research, role playing, visual art perception

## The arts combined

The fields devoted to the psychological study of the arts are very segregated into individual arts domains. Somebody who carries out research in music cognition, for example, tends not to be very interested in the aesthetic analysis of paintings, and vice versa. Likewise, someone who studies cognitive aspects of literature might not be all that interested in dance movement, and vice versa. The absence of crosstalk between arts domains is not terribly surprising given that all researchers are specialists who are linked to professional communities. However, there are definite drawbacks in not being able to think outside of one's own field of study; comparison is one of the most important means of generating knowledge.

As Brown (2022) wrote in *The Unification of the Arts*, “a comparative analysis of the arts provides greater insight into each artform than is possible by looking at artforms in isolation” (p. 23). It is clear that cross-arts comparisons are not very common in the scientific fields devoted to the psychology of the arts despite the insights that they could offer. Brown (2024) argued that “a key step toward achieving some level of unification of the arts is to consider the simplest level of coupling, namely two-art combinations” (p. 4). Along these lines, he presented a theoretical discussion of the following combination: songs with words, music in film, dances with music, and danced narratives.

This Research Topic attempted to stimulate thinking about the interconnections among the arts. In its announcement at *Frontiers in Psychology*, it called for the study of the following possible connections between art forms: music and speech/language/poetry; dance and music; film and music, including music video and video games; dancing and acting/miming/storytelling; theatre and literature; visual art and literature; and visual art

and the performing arts. Which of these connections were actually examined in the papers that are now published as part of this Research Topic?

## The scope of the Research Topic

Spence and Di Stefano, in a review of the combinations of auditory and visual modalities in visual art, highlight the distinction between the literature on cross-modal matching—that is, at the level of the stimulus—and the literature on cross-modal effects, that is, at the level of the art perceiver. They present a framework that is also useful for interrogating the levels at which the authors in this Research Topic looked for connections between the arts. For example, in their study on color perception in timbre-color synesthetes, Reuter et al. investigate the combination of auditory and visual modalities at the level of the stimulus. In contrast, auditory and visual modalities are combined at the level of behavior in the article by Zhang et al.

In three articles in this Research Topic, researchers illustrate links between music and language, but they do so at different levels: at the level of the stimulus and its processing (Karbanova and Cui), at the levels of the brain and learning (Cui et al.), and ultimately at the level of aesthetic appraisal (Nemestothy and Reiterer). However, Cui et al. show that the connections between music and language are not always found at the level of the brain when examining how it engenders learning. As Nemestothy et al. argue in a review on phonaesthetics, the study of the neural underpinnings of aesthetic pleasure may serve as a springboard for further research on arts combinations. A connection can be made, for example, to the study of aesthetic appraisal in visual art, and more specifically in abstract art, as examined by Zhou and Leder in this Research Topic.

The combination of music and dance is not often studied in their combined form, despite the clear connections between these domains. In this Research Topic, however, Marin-Bucio et al. do so at the level of learning, specifically in the learning of Malian djembe dance improvisation. Upham et al. study dancing at the level of physiology and behavior, while investigating the role of laughter in different dance practices.

A great number of possible combinations of the arts remain underexamined in this Research Topic and in research in general, most especially theatre. A first foray is made in a paper by Brown, in which the author argues that theatrical role playing is an evolved communicative behavior reflecting the contributions of both gestural and vocal learning, afforded perhaps by the extensive expansion of the arcuate fasciculus during human evolution. It seems paradoxical that theatre would be the least studied of the arts domains in cognitive psychology and cognitive neuroscience, given that it has the strongest resemblance to everyday social interaction. After all, a play is basically a group of people having conversations with one another. It is perhaps this “proximity to the ordinary” that makes theatre less appealing to researchers, compared to more art-specific functions like musical harmony, abstraction in visual art, or entrainment of a body movement to a regular beat.

## Outlook

The overall aim of this Research Topic was to develop scientific models that are true to the richly integrated nature of the arts in human life. The articles presented here not only provide a template for studying the Arts Combined at different levels, but they also provide a template for studying them with different methodologies: from structural analyses of stimuli, diffusion-weighted imaging of white-matter tracts in the brain, perception experiments, analyses of piano performances and of dance videos, to autoethnography and even the study of art-like behaviors in non-human species (e.g., birdsong). Further investigation into the cognitive, neural, and evolutionary facets of the Arts Combined will require crosstalk between the scientific disciplines devoted, respectively, to music, dance, theatre, literature, and visual art, and the marriage of their various methodologies.
